# Intermittent Fasting and the Gut Microbiota: Mechanisms Linking Microbial Remodeling to Metabolic and Immune Regulation

**DOI:** 10.3390/nu18162657

**Published:** 2026-08-14

**Authors:** Natalia Diaz-Garrido, Alejandro Regaldiz, Sebastián Zagmutt, Pedro Cisternas, Marianela Bastías-Pérez, Adrián Cortés-Martín

**Affiliations:** 1 Universidad Católica del Maule, Facultad de Ciencias de la Salud, Escuela de Nutrición y Dietética, Curicó 3340000, Chile; npdiaz@ucm.cl; 2 Programa de Doctorado en Biociencias Moleculares, Facultad de Ciencias de la Vida, Universidad Andres Bello, Santiago 8370186, Chile; regaldis.alejandro@gmail.com; 3 Department of Biomedical Sciences, Faculty of Medicine and Health Sciences, Universitat Internacional de Catalunya, 08195 Sant Cugat del Vallès, Spain; szagmutt@uic.es; 4 Núcleo de Investigación en Nutrición y Ciencias Alimentarias (NINCAL), Facultad de Salud y Ciencias Sociales, Universidad de Las Américas, Santiago 7500658, Chile; pcisternasf@udla.cl; 5 Centro de Investigación en Ciencias Biológicas y Químicas (CICBQ), Facultad de Medicina Veterinaria y Agronomía, Universidad de Las Américas, Santiago 7500658, Chile; 6 Centro de Investigación Biomédica en Red (CIBER) de Fisiopatología de la Obesidad y Nutrición (CIBEROBN), Instituto de Salud Carlos III (ISCIII), 28029 Madrid, Spain; 7 Sport and Health University Research Institute (iMUDS), University of Granada, 18016 Granada, Spain

**Keywords:** intermittent fasting (IF), gut microbiota, microbiome remodeling, immune regulation, metabolic health, circadian rhythms, short-chain fatty acids (SCFAs), prebiotics and probiotics

## Abstract

Intermittent fasting (IF) has gained increasing attention as a dietary strategy to improve metabolic health and prevent cardiometabolic disorders. Accumulating evidence suggests that modulation of the gut microbiota may represent one of the mechanisms underlying the physiological benefits of IF. This review summarizes the current knowledge on the mechanisms by which IF modulates gut microbial ecology and how these changes influence host metabolic and immune functions. We examine the effects of IF on gut microbiota diversity and composition, highlighting shifts in key microbial taxa associated with metabolic regulation. In addition, we discuss how fasting-induced microbial remodeling affects microbiota-derived metabolites, including short-chain fatty acids and bile acids, which play central roles in energy homeostasis, intestinal barrier integrity, and inflammatory signaling. Increasing evidence indicates that IF interacts with circadian rhythms, influencing both microbial oscillations and host metabolic pathways that coordinate nutrient sensing and energy metabolism. Furthermore, we explore the bidirectional crosstalk between the gut microbiota and the intestinal immune system, emphasizing that fasting-driven microbial changes may modulate inflammatory responses, epithelial barrier function, and immune cell activity. Finally, we discuss nutritional strategies that may enhance the beneficial effects of IF, including the incorporation of prebiotics, dietary fiber, and probiotic supplementation, to promote microbial diversity and functional resilience. Collectively, these findings support a model in which IF acts as a key modulator of the gut microbiota–immune–metabolic axis. Future integrative studies combining gut microbiome, metabolomic, and immunological approaches are needed to better understand these interactions and optimize microbiota-targeted dietary interventions.

## 1. Introduction

Intermittent fasting (IF) has emerged as a promising nutritional strategy for the prevention and management of obesity [1]. A growing body of evidence suggests that IF may provide multiple health benefits, including improved weight regulation, glucose homeostasis, reduced oxidative stress, and improved cardiometabolic health [2]. As a form of temporal restriction of food intake that is often, but not necessarily, associated with reduced energy intake, IF is increasingly recognized as a practical and flexible alternative to traditional continuous calorie restriction (CR). Recent studies have advanced our understanding of the physiological mechanisms underlying IF, highlighting its capacity to activate protective metabolic pathways that may contribute to obesity prevention and reduce the risk of obesity-related metabolic disorders [3].

Building on these insights, increasing attention has been directed toward the gut microbiota as a potential mediator of metabolic health [4]. The gut microbiota, composed of trillions of microorganisms residing in the gastrointestinal tract, plays an important role in energy homeostasis, nutrient metabolism, and immune regulation [5]. Accumulating evidence indicates that dietary patterns, particularly IF, can modulate the composition, diversity, and metabolic activity of the gut microbiota, especially under conditions of dysbiosis, which are commonly associated with obesity and metabolic diseases [6,7,8].

By promoting a more balanced microbial ecosystem, IF has been shown to reduce intestinal permeability, thereby attenuating systemic inflammation and lowering the risk of inflammation-associated conditions, including inflammatory bowel disease, metabolic syndrome, and autoimmune disorders [9,10,11]. These effects contribute to improved physiological homeostasis and overall health. Emerging clinical studies suggest that IF can reshape the composition and functional capacity of the gut microbiota, with such microbial alterations being closely associated with improvements in cardiometabolic risk factors [12,13,14].

Among the proposed mechanisms underlying these benefits, particular attention has been directed toward microbiota-derived metabolites, especially short-chain fatty acids (SCFAs), which play a central role in regulating host energy metabolism, immune responses, and intestinal barrier integrity [15,16,17]. Furthermore, IF has been reported to modulate tissue-specific immunometabolic responses that regulate metabolic flexibility and inflammatory signaling across key metabolic organs, including the liver, adipose tissue, skeletal muscle, and immune cells [12,18]. These effects may be partly mediated by the gut microbiota and are closely linked to circadian regulation, as IF can modulate circadian rhythms that govern a broad range of physiological processes involved in metabolic health and homeostasis [19,20].

Despite the growing body of evidence describing the beneficial effects of IF, the molecular mechanisms linking fasting-induced microbial remodeling to host metabolic and immune regulation remain incompletely understood. Although several recent reviews have summarized the effects of IF on gut microbiota composition and metabolic outcomes, comparatively less attention has been given to the mechanistic interactions connecting microbial community dynamics with microbiota-derived metabolites, circadian regulation, mucosal immune responses, and immunometabolic signaling. Integrating these interconnected pathways is essential to better understand how IF translates microbial adaptations into systemic metabolic benefits. Therefore, this review provides a comprehensive mechanistic framework that synthesizes current evidence on how IF modulates gut microbiota composition and function, and critically discusses the roles of microbial metabolites, circadian biology, and immune regulation in mediating the metabolic effects of fasting, with particular emphasis on obesity and type 2 diabetes mellitus (T2DM).

## 2. Intermittent Fasting: A Strategy for Shaping the Gut Microbiota and Obesity Control

### 2.1. Role of the Diet in Shaping Gut Microbiota

The gut microbiota constitutes a dynamic ecosystem that is closely involved in human health and disease [21,22]. It contributes to the digestion of complex nutrients, synthesis of essential vitamins, and maturation and modulation of the host immune system [23]. Owing to its dynamic nature, its composition and functionality are influenced by factors such as age, genetics, the environment, lifestyle, and diet [24,25].

Nutritional interventions are one of the main determinants shaping the gut microbiota composition and can induce detectable changes in microbial community structure within 24 h [26,27,28]. Although dietary modifications appear to produce transient shifts in microbial composition, the duration required to elicit permanent changes in core microbial profiles remains unclear. David et al. demonstrated that the gut microbiota can be modified by short-term dietary interventions; however, these changes persist for only a few days, suggesting that the microbiota resists certain external perturbations to maintain its inherent structure [27]. Conversely, Sonnenburg et al. reported in a multigenerational mouse model that prolonged consumption of a low-fiber diet can lead to the progressive loss of specific commensal taxa across generations, with some alterations becoming difficult to reverse through dietary intervention alone [29].

Additionally, the composition and quality of the diet shape the gut microbiota, influencing its composition and function, thereby affecting host-microbe interactions [30]. It has been reported that interindividual variability depends substantially on diet, which accounts for approximately 50% of the variability observed in mice and 20% in humans, underscoring the importance of dietary habits in modulating the gut microbiota [31]. Beyond nutritional interventions, feeding patterns have been shown to restructure gut microbial communities [32]. An unbalanced diet maintained over time, especially when accompanied by an unhealthy lifestyle, can leave detectable signatures in the gut microbiota. For example, individuals with obesity or overweight status generally exhibit reduced microbial diversity [33]. This pattern may be largely attributable to the competitive metabolic advantage conferred upon these taxa by a diet rich in simple sugars, such as sucrose-rich diets, which facilitates their growth [34].

### 2.2. Intermittent Fasting: A Metabolic Strategy for Obesity Control

The obesity epidemic has prompted the development of novel and effective dietary intervention strategies. IF has emerged as one of the most popular strategies based on temporal restriction of food intake, with reported benefits for weight management and cardiometabolic health [1]. IF is a dietary pattern characterized by alternating periods of fasting and ad libitum food intake according to a regular schedule. Although IF primarily restricts the timing of food intake rather than energy intake, it is often associated with an unintentional reduction in energy intake [35].

Mechanistically, IF activates a metabolic switch from glucose utilization to fatty acid and ketone utilization as energy sources, representing an evolutionarily conserved metabolic response [36]. Through these metabolic adaptations, IF may offer an effective strategy to restore metabolic flexibility, enhance insulin sensitivity, and promote a healthier body composition [37].

Three main IF protocols have been extensively studied: Alternate Day Fasting (ADF), Periodic Fasting (PF), and Time-Restricted Eating (TRE) (Table 1) [38]. ADF involves alternating a “feed day” with a “fast day,” during which energy intake is ad libitum for 24 h on feed days and severely restricted on fast days [39]. PF consists of two fasting days and five feeding days per week, and is commonly referred to as the 5:2 diet. Fasting may occur on consecutive or nonconsecutive days, whereas feeding days allow ad libitum caloric intake [40]. TRE is a less restrictive approach in which individuals consume all meals within a defined daily time window, with the most common being a 16:8 schedule (16 h of fasting followed by an 8 h eating period) [41].

Clinical evidence suggests that IF confers a wide range of health benefits, including reductions in body weight and adiposity, lower blood pressure, improvements in glycemic control, and a more favorable cardiometabolic risk profile [3,42]. Conversely, some studies have indicated that IF may not be superior to conventional energy restriction in improving cardiovascular and metabolic outcomes [43,44,45]. More recent investigations in populations with overweight and obesity have proposed that intermittent energy restriction, involving 2–3 days of periodic fasting per week, results in greater weight loss and reduced body fat compared with continuous energy restriction [46,47]. Overall, reported effects vary considerably depending on the type of IF implemented, intervention duration, and baseline characteristics of the study population [48,49,50].

## 3. Intermittent Fasting and Its Influence on the Human Gut Microbiota

**Figure 1:**
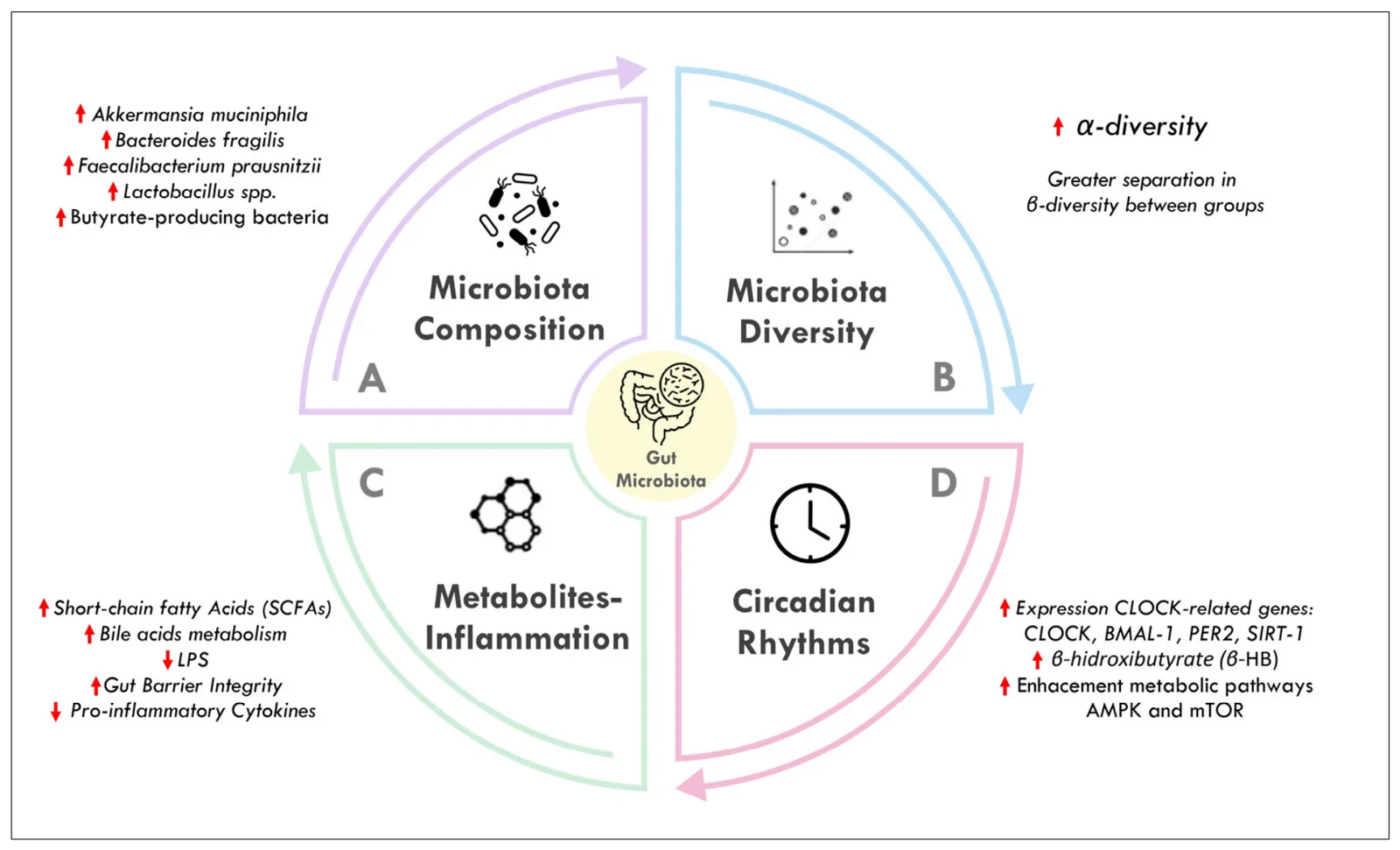
Schematic representation of the relationship between gut microbiota and intermittent fasting. (**A**) Intermittent fasting-induced changes in gut microbiota composition, including increased abundance of *Akkermansia muciniphila*, *Faecalibacterium prausnitzii*, *Lactobacillus* spp., and butyrate-producing bacteria, with reduced levels of *Bacteroides fragilis*. (**B**) Enhanced gut microbial diversity, reflected by higher α-diversity and greater separation in β-diversity between groups. (**C**) Modulation of metabolite production and inflammatory responses, characterized by increased short-chain fatty acids (SCFAs), improved bile acid metabolism and gut barrier integrity, together with reduced pro-inflammatory cytokines. (**D**) Regulation of circadian rhythms and metabolic pathways through the expression of *CLOCK*-related genes (*CLOCK*, *BMAL-1*, *PER2*, and *SIRT-1*), enhanced enteroendocrine signaling, and activation of AMPK and mTOR pathways.

Several studies have indicated that IF may exert an impact on the gut microbiota by modulating microbial diversity, and the abundance of specific bacterial species [51]. These effects may help mitigate some of the gut microbial alterations associated with high-fat diets [52]. In the following section, we examine the effects of IF on the gut microbiota diversity, composition, metabolite production, and circadian rhythms (Figure 1).

For clarity and ease of comparison, the evidence discussed in this section is summarized in Table 2 (human studies) and Table 3 (animal studies). These tables systematically compare the studies included according to the experimental model, IF protocol, intervention duration, and the principal taxonomic and functional changes in the gut microbiota.

### 3.1. Effects on Gut Microbiota Diversity

Alpha diversity is one of the most used metrics to characterize gut microbial communities and is often used as an indicator of gut microbiota health [53]. Alpha diversity describes the species richness, evenness, or diversity within a sample [54]. In general, ecosystems with high diversity indicate greater stability and ecological function [55]. Lower alpha diversity has been observed in several acute and chronic diseases [56]. In contrast, beta diversity assesses differences in microbial community composition between two or more samples or communities [57].

Studies have explored the effects of TRE on microbial diversity in both healthy individuals and individuals with overweight or obesity. In healthy individuals, microbial diversity was significantly higher in the TRE group than in the control group after 25 days of 16:8 intervention [58]. However, studies conducted in individuals with overweight or obesity reported no significant changes in microbial diversity from baseline or differences compared with control conditions after 12 weeks of TRE [7,59,60]. Dote-Montero et al. found no significant differences in fecal microbiota composition or diversity between early, late, or self-selected TRE and usual care, suggesting that neither the implementation nor the timing of an 8-h eating window induced detectable changes in the fecal microbiota over 12 weeks [59].

**Table 2:** Human studies evaluating the effects of intermittent fasting on gut microbiota and immune responses.

Reference	Country	Study Design	Sample Size	Participants	IF Protocol	Duration	Control	Gut Microbiota Diversity	Taxonomic/Compositional Changes	Functional Changes	Immune Changes
**Remely et al., 2015** [61]	Austria	Single-arm pilot with sequential fasting, laxative, and probiotic intervention	13	Adults with overweight (mean age, 53.3 years; mean BMI, 28.1 ± 3.5 kg/m^2^)	One-week supervised Buchinger fasting with laxative, followed by a probiotic/prebiotic formula	1 week fasting + 6 weeks probiotic intervention	No parallel control	Microbial diversity increased over the full intervention period	Increased *Faecalibacterium prausnitzii*, *Akkermansia* spp., and Bifidobacteria; Enterobacteria and Lactobacilli increased during fasting and subsequently declined	Not assessed	Not assessed
**Cignarella et al., 2018** [62]	United States	Randomized controlled pilot trial	17	Adults with relapsing-remitting multiple sclerosis (BMI ≥ 23 kg/m^2^)	Alternate-day fasting	15 days	Ad libitum diet	Gut microbiota shifted toward a profile partially resembling that observed in the murine IF model	Partial recapitulation of protective microbial changes observed in mice	↓ Leptin; no significant clinical or neurological improvements over the short intervention	Not assessed
**Mesnage et al., 2019** [63]	Germany	Single-arm longitudinal natural experiment	15	Relatively healthy men aged 18–70 years; BMI 20–32 kg/m^2^	Buchinger periodic fasting (~250 kcal/day) with an enema every 2 days	10 days, with refeeding and 3-month follow-up	No parallel control; participants served as their own controls	No major diversity change emphasized; fasting-induced community shifts largely reversed after 3 months	Decreased Lachnospiraceae and Ruminococcaceae; increased Bacteroidetes, Proteobacteria (*Escherichia coli*, and *Bilophila wadsworthia)*	Fecal SCFAs unchanged at the end of fasting; taxonomic shifts associated with serum glucose and fecal BCAAs; metabolic switch toward fatty acids and ketones	IL-6, IL-10, IFN-γ, and TNF-α increased during food reintroduction rather than during fasting
**Özkul et al., 2019** [64]	Turkey	Uncontrolled natural-event preliminary study	9	Healthy, normal-weight adults aged 31–56 years (7 women, 2 men)	Ramadan fasting (~17 h/day from sunrise to sunset)	29 days	No control; pre–post comparison	Not assessed by community sequencing (targeted qPCR only)	Increased *Akkermansia muciniphila* and *Bacteroides fragilis* group; no significant changes in *Faecalibacterium prausnitzii*, *Bifidobacterium* spp., *Lactobacillus* spp., or Enterobacteriaceae	Not assessed	Not assessed
**Özkul et al., 2020** [65]	Turkey	Uncontrolled natural-event pilot study	9	Healthy, normal-weight adults aged 31–56 years (7 women, 2 men)	Ramadan fasting (~17 h/day)	29 days	No control; pre–post comparison	Observed OTU richness increased; Shannon index and phylogenetic diversity unchanged; unweighted UniFrac β-diversity changed significantly	Increased *Butyricicoccus* spp., *Bacteroides* spp., *Faecalibacterium* spp., *Roseburia* spp., *Allobaculum* spp., *Eubacterium* spp., *Dialister* spp., and *Erysipelotrichi* spp.; *Butyricicoccus pullicaecorum* was the most discriminative species	Functional capacity not directly measured; several enriched taxa were associated with butyrate production	Not assessed
**Gabel et al., 2020** [60]	United States	Single-arm pilot; secondary analysis	14 with complete stool sampling	Adults aged 25–65 years with obesity (BMI 30–45 kg/m^2^), non-diabetic	Daily 16:8 TRE; ad libitum eating from 10:00 to 18:00	12 weeks	No parallel control	OTU richness, Shannon diversity, and phylogenetic diversity unchanged	No significant changes in Firmicutes, Bacteroidetes, or other major phyla; overall composition unchanged	Not assessed	Not assessed
**Zeb et al., 2020** [66]	China	Multicenter controlled human intervention	80 (TRE, 56; non-TRE, 24)	Healthy young adult men	Daily 16-h fast with an 8-h eating window (19:30–03:30)	25 days	Usual diet without time restriction	Microbial richness increased significantly in the TRE group	Enrichment of Prevotellaceae and Bacteroidaceae; compositional differences compared with controls	Microbial richness was positively associated with SIRT1 and circadian-gene expression and with improved metabolic markers	Reduced IL-1β and TNF-α gene expression and circulating levels
**Zeb et al., 2020** [58]	China	Cross-sectional controlled comparison after intervention	30 (TRE, 15; non-TRE, 15)	Healthy sedentary men aged 18–30 years	Daily 16-h fasting for 25 days; habitual foods during the eating window	25 days	Non-TRE habitual diet	Microbial abundance/richness appeared higher in TRE; no longitudinal baseline sampling	TRE enriched *Prevotella_9*, *Faecalibacterium*, and *Dialister*; non-TRE enriched *Prevotella_7*, *Alloprevotella*, and *Prevotella_2*; Bacteroidetes predominated in TRE	Nutrient–microbiota correlations were reported; PUFA and vitamin D were positively associated with Firmicutes; predicted LPS biosynthesis was reduced	Not assessed
**Stanislawski et al., 2021** [67]	United States	Ancillary analysis of a randomized controlled trial (parallel-group)	59 (IMF, 34; DCR, 25)	Adults aged 18–55 years with overweight/obesity (BMI 27–45 kg/m^2^)	Intermittent fasting: 80% energy restriction on 3 non-consecutive days/week; ad libitum intake on remaining days	12 months	Daily caloric restriction (matched weekly energy deficit)	Overall microbial diversity changed during intervention; baseline diversity associated with weight-loss response	Between-group differences in *Akkermansia* spp. abundance; microbiota composition associated with weight and waist circumference	Not assessed	Not assessed
**Guo et al., 2021** [12]	China	Randomized controlled parallel-group clinical trial	39 (IF, 21; control, 18)	Adults aged 30–50 years with metabolic syndrome	Modified 5:2 IF: 75% energy restriction on 2 non-consecutive days/week; ad libitum intake on the other 5 days	8 weeks	Habitual control diet without specific dietary instructions	Shannon α-diversity unchanged; gut community structure/β-diversity changed significantly	Twenty-three taxa changed; enrichment of SCFA-associated taxa, including Ruminococcaceae- and *Roseburia*-related bacteria	Increased fecal SCFAs and carbohydrate-metabolism pathways; decreased circulating LPS	Reduced sCD40L and leptin; increased adiponectin; IL-6 and TNF-α were unchanged
**Maifeld et al., 2021** [68]	Germany	Randomized controlled substudy with multi-omics and deep immunophenotyping	71 at baseline (fasting + DASH, 35; DASH, 36)	Adults with hypertensive metabolic syndrome (mean age ~60 years; BMI ~33–34 kg/m^2^)	Five-day modified fast followed by a modified DASH diet	5-day fast + 3-month DASH follow-up	Modified DASH diet alone	Species richness/Shannon α-diversity unchanged; within-person microbial composition shifted during fasting and partially reverted after refeeding	Multiple transient taxonomic shifts; response-associated taxa included Desulfovibrionaceae, Hydrogenoanaerobacterium, *Akkermansia* spp., and Ruminococcaceae	Altered microbial gene modules, including pathways related to SCFA production; microbiome signatures predicted sustained blood-pressure response	Reduced CD3+/CD4+ T cells and B cells; increased monocytes, γδ-T cells, and plasmacytoid dendritic cells; reduced pro-inflammatory Th17, Th1, and MAIT-cell responses
**Xie et al., 2022** [69]	China	Randomized controlled three-arm, outcome-assessor-blinded trial	82 completed (eTRE, 28; mTRE, 26; control, 28)	Healthy adults without obesity	eTRE: ≤8-h eating window between 06:00 and 15:00; mTRE: ≤8-h window between 11:00 and 20:00	5 weeks	Ad libitum eating without time restriction	eTRE increased gut microbial α-diversity; mTRE did not show a comparable improvement	eTRE produced broader community remodeling than mTRE; multiple differential taxa were reported	Microbiome changes were associated with altered circadian and adipokine rhythms; SCFAs were not directly measured	eTRE reduced TNF-α and IL-8 versus control; hsCRP was unchanged
**Khan et al., 2022** [70]	Pakistan	Single-arm pre–post clinical intervention	45 (31 men, 14 women)	Healthy adults aged 18–35 years across lean, normal-weight, and overweight/obesity phenotypes	16:8 IF for 26 days; ≥16 h abstention and an 8-h ad libitum eating window	26 days	No parallel control; baseline comparison	Overall α-diversity/evenness increased, with phenotype-dependent responses; β-diversity changed	Increased *Lactobacillus* spp. and *Bifidobacterium* spp.; pathogenic taxa generally decreased; marked phenotype- and sex-specific taxonomic shifts	Shotgun metagenomic profile changed; culturable aerobic bacteria decreased and fungal counts increased	CRP remained negative/not elevated; no cytokine panel was performed
**Mohr et al., 2022** [71]	United States	Randomized exploratory secondary analysis comparing two active interventions	20 (IF1-P, 10; IF2-P, 10)	Healthy sedentary/lightly active adults with overweight or obesity	IF1-P: one 36-h fast/week; IF2-P: two consecutive fasting days (60 h total)/week; protein pacing on feeding days	4 weeks	Active comparator: one-day versus two-day IF; no non-fasting group	α-diversity unchanged; β-diversity changed over time in both groups, with no between-group difference	IF1-P increased Ruminococcaceae *Incertae Sedis* and *Eubacterium fissicatena* group; IF2-P increased Ruminococcaceae *Incertae Sedis* and decreased *Eubacterium ventriosum* group	Fecal SCFAs unchanged; IF2-P altered the plasma metabolome, including increases in serine, TMAO, citrate, isocitrate, and glucuronic acid	Not assessed
**Ferrocino et al., 2022** [7]	Italy	Non-randomized preference-based controlled real-life intervention	49 completed (TRE, 25; TUE, 24)	Adults aged 18–75 years with obesity (BMI 30–<45 kg/m^2^) and low physical activity	TRE: feeding window <12 h/day; same hypocaloric diet in both groups	12 weeks	Time-unrestricted eating (>12 h/day) with matched caloric restriction	No meaningful between-group differences in α- or β-diversity	Overall composition was largely unchanged; within TRE, Lachnospiraceae, Parasutterella, and Romboutsia increased	Not assessed	Not assessed
**Hu et al., 2023** [72]	China	Single-arm prospective intervention study	72	Adults aged 18–55 years with BMI 18.5–30 kg/m^2^ (normal weight to obesity), free of metabolic diseases	Modified 5:2 intermittent fasting: two non-consecutive fasting days/week (500–600 kcal/day, ~25% of habitual energy intake); ad libitum intake on the remaining five days	3 weeks	No parallel control; pre–post comparison	Diversity indices were not the primary endpoint; marked compositional remodeling after IF	Enrichment of *Parabacteroides distasonis*, *Bacteroides thetaiotaomicron*, *Parabacteroides merdae*, *Bacteroides uniformis* and *Fusicatenibacter saccharivorans*; decreased *Agathobacter rectalis*, *Clostridium*, *Coprococcus* and *Enterocloster*; IF-enriched species inversely correlated with LDL-C and atherosclerosis index	Shotgun metagenomics revealed enrichment of carbohydrate-active enzyme (CAZyme) genes and pathways related to carbon metabolism, citrate cycle, pentose phosphate pathway, succinate production and glutamate fermentation, suggesting enhanced microbial metabolic capacity and SCFA-generating potential	Not assessed
**Batitucci et al., 2024** [73]	Brazil	Randomized three-arm clinical trial	36 completed (IF + HIIT, 15; HIIT, 11; IF, 10)	Sedentary women aged 18–40 years with obesity (BMI 30–40 kg/m^2^) and no comorbidities	Modified 5:2 IF on 2 non-consecutive days/week; 25% of energy needs; 6-h feeding/18-h fasting on IF days; with or without HIIT	8 weeks	HIIT-only active comparator	No significant α- or β-diversity changes attributable to IF or HIIT	No significant changes in overall taxonomic composition or predicted functional profile	Fecal acetate increased only in the exercise group; propionate and butyrate were unchanged	No significant effects on IL-15, IL-10, TNF-α, resistin, adipsin, fetuin-A, or CRP
**Dote-Montero et al., 2026** [59]	Spain	Multicenter parallel four-arm randomized controlled trial; secondary microbiota analysis (preprint)	197 randomized (UC, 49; early TRE, 49; late TRE, 52; self-selected TRE, 47)	Men and women aged 30–60 years with overweight/obesity, abdominal obesity, and ≥1 cardiometabolic risk factor	Eight-hour eating window: early, late, or self-selected TRE; all combined with Mediterranean diet-based usual care	12 weeks	Mediterranean diet-based usual care alone	No between-group differences in Shannon, Simpson, Chao1, observed features, or Bray–Curtis β-diversity	No significant between-group changes in taxonomic composition at phylum or genus level	Not assessed	Not assessed

Abbreviations: BCAA, branched-chain amino acid; BMI, body mass index; CRP, *C*-reactive protein; DASH, Dietary Approaches to Stop Hypertension; eTRE, early time-restricted eating; HIIT, high-intensity interval training; IF, intermittent fasting; IFN-γ, interferon gamma; IL, interleukin; LPS, lipopolysaccharide; MAIT, mucosa-associated invariant T cell; mTRE, mid-day time-restricted eating; OTU, operational taxonomic unit; SCFA, short-chain fatty acid; TMAO: trimethylamine *N*-oxide; TNF-α: tumor necrosis factor alpha; TRE, time-restricted eating; TUE, time-unrestricted eating; UC, usual care.

**Table 3:** Preclinical animal studies evaluating the effects of intermittent fasting on gut microbiota, microbial metabolites, intestinal barrier, and immune modulation.

Reference	Animal Model	Disease Model	Diet	IF Protocol	Duration	Microbiota Alterations	Microbial Metabolites	Gut Barrier	Immune Modulation
**Godínez-Victoria et al., 2014** [74]	Male BALB/c mice; 8 weeks old; *n* = 24 (12/group; 6/time point)	Sublethal oral *Salmonella Typhimurium* infection	NIH-31 standard chow	Alternate-day fasting for 12 weeks before infection; animals fed ad libitum after infection	12 weeks; outcomes at 7 and 14 days post-infection	Microbiota composition was not characterized; lower fecal, Peyer’s patch, liver, and spleen Salmonella loads indicated enhanced pathogen clearance	Not assessed	Not assessed	Increased total and Salmonella-specific intestinal SIgA, IgA+ plasma cells, and mucosal pIgR, α-chain, J-chain, Pax-5 and cytokine transcripts; lower systemic and intestinal bacterial burden
**Zarrinpar et al., 2014** [52]	Male C57BL/6J mice; 12 weeks old; *n* = 24/condition (microbiome time-series *n* = 18/condition)	Diet-induced obesity and metabolic dysfunction	Normal chow or HFD	TRE: HFD available for 8 h during dark phase (ZT13-ZT21) versus ad libitum HFD or chow	8 weeks	HFD dampened diurnal microbial oscillations; TRE partially restored cyclic OTUs and increased temporal diversity; altered *Lactobacillus* spp., *Lactococcus* spp. and obesity-associated taxa	Metagenomic functional potential oscillated with feeding/fasting; TRE restored rhythms in pathways related to nutrient and energy metabolism	Not assessed	Not assessed
**Lara-Padilla et al., 2015** [75]	Male BALB/c mice; 8 weeks old; *n* = 6/group (4 groups)	Intense physical stress induced by forced swimming	NIH-31 standard chow	Alternate-day fasting, followed by a final 5.5-h forced-swimming stress challenge	12 weeks	Not assessed	Not assessed	Regional modulation of mucosal homeostasis; intestinal pIgR expression and SIgA responses differed between duodenum and ileum	IF plus intense stress differentially modulated SIgA, IgA+ plasma cells, pIgR/IgA-chain transcripts, TNF-α, IL-6, IFN-γ, IL-2, IL-4, IL-10 and TGF-β; corticosterone increased
**Li et al., 2017** [11]	Male C57BL/6J mice; chow and HFD models; group sizes varied by experiment	Diet-induced obesity, insulin resistance and hepatic steatosis	Chow or HFD	Every-other-day fasting (24-h fasting alternating with 24-h feeding)	15 cycles (30 days); additional mechanistic and transplantation experiments	EODF reshaped community structure; increased Firmicutes and enriched taxa including *Lactobacillus* spp. and *Butyricicoccus* spp.; microbiota depletion blocked, and fecal transfer reproduced, WAT beigeing	Increased acetate and lactate; microbial functional pathways linked to carbohydrate fermentation and energy metabolism were altered	Not assessed	Microbiota-dependent metabolic effects; direct immune-cell profiling was not performed
**Cignarella et al., 2018** [62]	Female C57BL/6 mice; EAE experiments typically *n* = 10/group	Experimental autoimmune encephalomyelitis (multiple sclerosis model)	Standard chow	Alternate-day fasting for 4 weeks before EAE induction and continued during disease	4 weeks before immunization; continued through EAE	Increased bacterial richness and altered β-diversity; enrichment of Lactobacillaceae, Bacteroidaceae and Prevotellaceae; fecal microbiota transfer from IF donors reduced EAE severity	Enhanced predicted antioxidant and microbial metabolic pathways; increased serum β-hydroxybutyrate and adiponectin, reduced leptin	Not assessed	Reduced IL-17A- and IFN-γ-producing CD4+ T cells and pathogenic cytokines; increased intestinal Treg cells and reduced Th17 cells; reduced CNS inflammation, demyelination and axonal damage
**Beli et al., 2018** [76]	Male *db*/*db* and *db*/*m* mice; four feeding/genotype groups; *n* varied by endpoint	Type 2 diabetes and diabetic retinopathy	Standard rodent diet; ad libitum or IF	Alternate-day fasting: 24-h fasting every other day	7 months	In *db*/*db* mice, IF increased Firmicutes and decreased Bacteroidetes and Verrucomicrobia; reduced *Bifidobacterium* spp., *Clostridium* spp. and *Bacteroides* spp.; β-diversity differed, richness/evenness largely unchanged	Increased secondary bile acids, especially TCDCA and TUDCA; reduced plasma peptidoglycan	Increased mucin-positive goblet cells and villus length; improved intestinal morphology	Reduced retinal TNF-α, leukocyte infiltration, macrophages/microglial activation and acellular capillaries; TGR5 pathway implicated
**Li et al., 2020** [77]	Male SPF C57BL/6J mice; 6 weeks old; *n* = 15/group	Healthy mice	Standard irradiated chow	Daily fasting for 12, 16 or 20 h; ad libitum control	30 days, followed by 30-day washout	α-diversity unchanged; β-diversity/composition altered by all fasting schedules. The 16-h regimen increased *Akkermansia* spp. and decreased Alistipes spp.; effects largely disappeared after washout	Not assessed	Not assessed	Not assessed
**Liu et al., 2020** [78]	Male *db*/*db* mice and *db*/*m* littermate controls; 3 months old; group sizes varied across assays	Type 2 diabetes-associated cognitive impairment	Standard chow	Alternate-day fasting at 24-h intervals	28 days	IF restructured the gut microbiota; antibiotic depletion partially abolished neuroprotection. Microbial taxa were linked to hippocampal genes, metabolites and cognition. Enrichment in *Lactobacillus* spp.	Increased/normalized SCFAs, 3-indolepropionic acid, serotonin and TUDCA; multi-omics supported a microbiota-metabolites-brain axis	Not assessed	Suppressed hippocampal NF-κB, JNK/p38 signaling and microglial activation.
**Deng et al., 2020** [79]	Male C57BL/6J mice; 3 weeks old; initial *n* = 60; after DIO induction *n* = 15/group	HFD-induced obesity	LFD (10% fat) or HFD (60% fat)	Alternate-day fasting: alternating 24-h feeding and 24-h fasting	30 days after 12-week of diet-induced obesity induction	Richness unchanged; tendency toward higher diversity; reduced Firmicutes/Bacteroidetes ratio and increased *Allobaculum* spp.	Reduced circulating LPS	Not assessed	Reduced metabolic endotoxemia
**Rust et al., 2023** [80]	Male C57BL/6 mice; 12 weeks old; *n* = 14/group	HFD-induced obesity/metabolic dysfunction	Control diet (16% fat) or HFD (48% fat)	HFD-TRE restricted to the 12-h dark phase (ZT12-ZT24)	12 weeks	No phylum-level changes; eight bacterial families altered by diet and/or feeding time; distinct taxon-lipid relationships	TRE increased fecal total fatty-acid excretion; attenuated HFD-induced reduction in total SCFAs; primary bile acids unchanged	Not assessed	Not assessed
**Chen et al., 2024** [81]	Male C57BL/6 mice; NCD and HFD-induced obesity models; sample sizes determined by power analysis	Diet-induced obesity and impaired adipose thermogenesis	Normal chow or HFD	Alternate-day fasting	30 days (NCD experiments 4 weeks; HFD induced for 12 weeks before IF)	Not assessed	Not assessed	Not assessed	Increased intestinal and plasma IL-22 and IL-22 + ILC3s; activated AhR signaling and ILC3 interactions with dendritic cells/macrophages; IL-22R deficiency attenuated WAT beigeing
**Fu et al., 2025** [82]	Male BALB/c mice; 4 weeks old; *n* = 5/group in main IF model	IF-induced colonic inflammation	Standard diet	TRE with an 8-h daily feeding window	6 weeks	Dysbiosis with increased pro-inflammatory/pathogenic taxa and decreased SCFA-producing/beneficial bacteria	Reduced indoleacrylic acid and other anti-inflammatory metabolites; increased pro-inflammatory metabolites; IAA supplementation reversed effects	Impaired mucus layer and tight-junction/barrier integrity; increased permeability and histological injury	Increased pro-inflammatory cytokines and Th17 differentiation; reduced immune balance. IAA reduced Th17 activation and increased IL-10
**Li et al., 2026** [83]	MPTP-induced PD mice fed casein or soy protein; microbiome *n* = 8/group; most tissue assays *n* = 5–7	MPTP-induced Parkinson’s disease	Casein-based or soy-protein-based diets	Daily TRE combined with casein or soy protein diet	4 weeks	Protein-dependent remodeling: TRE reduced *Allobaculum* spp. in casein-fed PD mice and increased *Akkermansia* spp. in soy-fed PD mice	TRE reduced BCAAs with casein and increased SCFAs with soy; Allobaculum/BCAAs impaired organoid barrier function	Restored ZO-1, Occludin and Claudin-1, particularly in casein-fed PD mice; soy diet preserved barrier integrity	Reduced astrocyte/microglial activation and neuroinflammation; stronger neuronal protection with soy protein plus TRE

**Abbreviations:** AhR, aryl hydrocarbon receptor; BCAA, branched-chain amino acid; DIO, diet-induced obesity; EAE, experimental autoimmune encephalomyelitis; EODF, every-other-day fasting; HFD, high-fat diet; IAA, indoleacrylic acid; IF, intermittent fasting; IFN-γ, interferon gamma; IL, interleukin; ILC3, type 3 innate lymphoid cell; LFD, low-fat diet; LPS, lipopolysaccharide; NCD, normal chow diet; PD, Parkinson’s disease; SCFA, short-chain fatty acid; SIgA, secretory immunoglobulin A; TCDCA, taurochenodeoxycholic acid; TGR5, G protein-coupled bile acid receptor 1; TNF-α: tumor necrosis factor alpha; TRE, time-restricted eating; TUDCA, tauroursodeoxycholic acid; WAT, white adipose tissue; ZT, Zeitgeber time.

In contrast, Xie et al. reported increased gut microbial diversity following a 5-week early TRE (eTRE), but not mid-day TRE, in healthy individuals without obesity [69]. Evidence from ADF interventions has also shown variable effects on microbial diversity. A three-month ADF intervention in individuals with overweight or obesity was associated with changes in alpha and beta diversity [67]. Similarly, Cignarella et al. found that ADF increased alpha bacterial diversity, which was inversely related to blood leptin levels [62]. However, no significant differences in terms of diversity were reported between the traditional energy restriction and the PF protocol (5:2 diet), which suggests that short-term IF regimens may have limited impact on gut microbiota in individuals with obesity [12,71,73]. Then, the effects of IF on gut microbial diversity may depend on the specific fasting protocol, intervention duration, and study population.

### 3.2. Effects on Gut Microbiota Composition

Several studies have investigated the effects of TRE on gut microbiota composition, although the reported findings are heterogeneous. In adults without obesity, TRE 16:8 protocol for 29 days has been associated with an increased relative abundance of *Bacteroides fragilis* and *Akkermansia muciniphila* [64,65]. In mice subjected to a six-hour TRE for one-month, increased levels of *A. muciniphila* and a decreased abundance of the genus *Alistipes* were reported [77]. Conversely, a study in women with obesity showed decreases in the relative abundance of Firmicutes, Actinobacteria, and Bacteroidetes, together with an increase in Proteobacteria in response to TRE [70]. Likewise, an increase in the abundance of *Lactobacillus* spp., *A. muciniphila*, *Faecalibacterium prausnitzii*, and *Enterobacteria* spp., was reported in adults with overweight and obesity after TRE intervention [61], whereas Gabel et al. found no changes in the relative abundance of Firmicutes and Bacteroidetes in individuals with obesity during a 16:8 intervention [60]. Several studies have also investigated the impact of TRE on butyrate-producing bacterial species. For example, Zeb et al. showed an increased abundance of butyrate-producing members of the Ruminococcaceae family following TRE 16:8 protocol for 25 days [66].

Regarding the ADF protocol 5:2, a human study reported increasing trends in the abundance of *F. prausnitzii*, *Lachnospiraceae incertae sedis*, and *Blautia* after 15 days of intervention [62]. Moreover, another study reported changes in the family Ruminococcaceae and the genus *Roseburia* after a 5:2 diet for 8 weeks, suggesting an alteration of the gut microbial community [12]. Mohr et al. found no differences in the abundance of taxa above the genus level after 4 weeks of intervention under 5:2 protocol. However, an increased abundance of *L. incertae sedis* was observed in the intervention groups [71]. *L. incertae sedis* comprises taxa associated with the production of butyrate and other SCFAs, which are considered important for gut health. Similarly, three months of periodic fasting increased the abundance of *Bacteroides* spp., *Alistipes* spp., and *A. muciniphila* [67]. In a Chinese cohort, the 5:2 protocol was also associated with an increased abundance of *Parabacteroides* spp. and *Bacteroides* spp., species that have previously been shown to exert beneficial effects on energy metabolism and metabolic outcomes [72]. Animal studies have further shown that one month of ADF increased the abundance of the families Lactobacillaceae, Bacteroidaceae, and Prevotellaceae in healthy mice [77]. In addition, both short-term (4 weeks) and long-term (7 months) ADF increased the abundance of *Lactobacillus* spp. and improved gut function and physiology in *db*/*db* mice [76,78].

Although both animal and human studies indicate that IF can modulate gut microbiota composition, the magnitude and direction of these changes differ considerably across experimental models. These discrepancies are likely explained by multiple biological and methodological factors rather than by intervention duration alone. Animal studies are typically conducted under highly controlled conditions, including standardized diets, homogeneous genetic backgrounds, controlled housing environments, and strict adherence to fasting regimens, allowing more consistent detection of microbiota alterations [84,85]. In contrast, human studies are inherently more heterogenous, with substantial interindividual variability in baseline microbiota composition, habitual dietary intake, medication use, lifestyle, age, and adherence to fasting protocols all which can influence microbial responses [25,86]. Moreover, differences in fasting duration, feeding schedules, degree of energy restriction, microbiome sequencing approaches, bioinformatic pipelines, sample collection timepoints, and the type of biological specimen analyzed (fecal versus mucosal samples) may further contribute to the inconsistent findings reported across studies, as fecal microbiota does not necessarily reflect microbial communities residing at the intestinal mucosal interface [87,88]. Therefore, extrapolation of these microbiota responses from animal models to humans should be interpreted with caution.

Overall, current evidence indicates that IF can modify gut microbiota composition; however, the reported microbial alterations differ substantially across studies. This heterogeneity likely reflects differences in IF protocols, host characteristics, microbiome sampling strategies (including fecal and mucosal sampling), sequencing methodologies, and analytical pipelines, highlighting the need for standardized study designs and functional microbiome analysis to facilitate comparisons across studies [6,84,85].

Additionally, although changes in microbial diversity and taxonomic composition provide valuable ecological insights, they do not necessarily reflect microbial functionality or predict clinical outcomes. Increasing evidence indicates that metabolic improvements following IF may occur despite minimal changes in α-diversity or bacterial abundance, suggesting that functional adaptations of the gut microbiome are likely to be more relevant than taxonomic alterations alone [87,89,90]. Consequently, integrating taxonomic analyses with functional approaches, including metagenomics, metatranscriptomics, metabolomics, and microbial metabolite profiling, will be essential to better understand the biological mechanisms through which IF influences host metabolism and immune regulation. The following section therefore focuses on microbiota-derived metabolites as key functional mediators of these effects.

### 3.3. Effects on Gut Microbiota-Derived Metabolites and Inflammatory Markers

IF has been associated with changes in microbiota-derived metabolites and host inflammatory markers. Metabolomic analyses of mice subjected to a TRE protocol for 12 weeks revealed significant metabolic alterations that may explain some of the observed phenotypic changes [80]. For example, TRE increased fecal fatty acid content, suggesting altered intestinal lipid absorption [80]. TRE has also been associated with changes in bile acid composition, modifying both primary and secondary bile acid pools across the intestine, liver, and circulation [91]. Bile acids function as signaling molecules that regulate lipid and cholesterol metabolism through receptors such as farnesoid X receptor (FXR) and Takeda G protein-coupled receptor 5 (TGR5) [92]. Although these findings suggest that TRE influences bile acid homeostasis, the available evidence does not allow these changes to be directly attributed to gut microbiota-mediated bile acid biotransformation. This is because microbial functional genes involved in bile acid metabolism, including BSH (bile salt hydrolase) and the BAI (bile acid-inducible) operon, were not evaluated [93,94]. Therefore, further mechanistic studies integrating metagenomics, metabolomics, and bile acid profiling are needed to clarify the contribution of the gut microbiota to fasting-induced alterations in bile acid metabolism. During fasting, several genes involved in lipid metabolism are upregulated. One of these is fasting-induced adipocyte factor (FIAF), an inhibitor of lipoprotein lipase (LPL), which promotes fatty acid oxidation and limits triglyceride storage [95]. Supporting this mechanism, studies with *Lactobacillus plantarum* MTCC5690 and *Lactobacillus fermentum* MTCC5689 reported increased FIAF expression, which may contribute to improved lipid metabolism and protection against diet-induced obesity [96].

Another key group of microbiota-derived metabolites comprises short-chain fatty acids (SCFAs), which play a central role in maintaining intestinal and metabolic homeostasis. Guo et al. reported that PF (5:2 diet) increased the relative abundance of SCFAs-producing species [12]. These compositional changes may enhance the capacity of the gut microbiota to produce SCFAs, which act as signaling molecules through the G protein-coupled receptors FFAR2 (GPR43) and FFAR3 (GPR41). Although both receptors respond to SCFAs, they differ in ligand affinity, tissue distribution, and downstream physiological effects [97,98]. FFAR2 is preferentially activated by acetate and propionate and is highly expressed in enteroendocrine and immune cells, whereas FFAR3 displays greater affinity for propionate and butyrate and is predominantly expressed in enteric neurons and other peripheral tissues [99,100]. An activation of these receptors contributes to the regulation of enteroendocrine hormone secretion, including glucagon-like peptide-1 (GLP-1) and peptide YY (PYY), thereby influencing satiety, glucose homeostasis, and energy metabolism [101,102,103,104]. Furthermore, SCFAs have emerged as key mediators of microbiota-host interactions owing to their essential role in maintaining intestinal barrier integrity, modulating intestinal immune responses and preserving gut microbial homeostasis [105,106]. In addition, SFCAs, together with other microbiota-derived neuroactive compounds such as GABA and serotonin, participate in the regulation of feeding behavior through gut–brain signaling pathways [102].

Beyond SCFAs, other microbiota-derived metabolites are increasingly recognized as potential mediators of the metabolic and immunological effects of IF. Among these, tryptophan-derived metabolites, including indole, indole-3-acetic acid (IAA), indole-3-propionic acid (IPA), and indole-3-aldehyde (IAId), have emerged as key signaling molecules linking microbial metabolism to host physiology [107]. These metabolites activate the aryl hydrocarbon receptor (AhR) and pregnane X receptor (PXR), thereby promoting epithelial barrier integrity, mucus production, antimicrobial peptide expression, and immune tolerance through the regulation of IL-22 and regulatory T-cell responses [108]. Although direct evidence remains limited, fasting-induced alterations in microbial composition and substrate availability may reshape tryptophan metabolism and downstream AhR signaling, representing a potential mechanism through which IF contributes to intestinal and systemic immune homeostasis. Likewise, growing evidence suggests that IF may influence microbial amino acid metabolism. In a murine Parkinson’s disease model, TRE combined with different dietary protein sources reshaped gut microbial metabolic pathways, including branched-chain amino acid (BCAA) metabolism, while promoting neuroprotective effects and improving gut microbiota function [83]. Since dysregulated BCAA metabolism has been associated with insulin resistance, obesity, and systemic inflammation, modulation of microbial BCAA biosynthesis may represent an additional mechanism through which IF contributes to metabolic homeostasis [109]. Another metabolite receiving increasing attention is trimethylamine *N*-oxide (TMAO), which is generated from dietary choline and L-carnitine through sequential microbial and hepatic metabolism [107,110]. Elevated circulating TMAO concentrations have been associated with insulin resistance, atherosclerosis, and cardiovascular disease, whereas recent clinical studies suggest that IF and TRE may reduce plasma TMAO concentrations, potentially contributing to improvements in cardiometabolic risk [111].

### 3.4. Effects on Gut Microbial Regulation of Circadian Rhythms

The temporal patterning of food intake constitutes a key regulatory factor of circadian rhythms, governing a broad spectrum of physiological processes fundamental to human health [20]. Although the mechanistic underpinnings of this association have not yet been fully elucidated, it is thought to involve bidirectional interactions with the gut microbiota, including changes in its composition and function [112].

The core circadian clock orchestrates biological processes in a 24-h cycle. It is primarily governed by the transcription factors *CLOCK* (circadian locomotor output cycles kaput) and *BMAL1* (brain and muscle aryl hydrocarbon receptor nuclear translocator protein 1), which are expressed in peripheral organs (liver, heart, lungs, and kidneys) [51]. These transcription factors drive the transcription and translation of clock-controlled genes, including *PER* (Period) and *CRY* (Cryptochrome), which participate in a transcriptional-translational feedback loop that further inhibits *CLOCK*-*BMAL1*-mediated transcription [113]. In turn, the clock is regulated by mechanisms that modulate the activity of nuclear receptors, such as SIRT1 (sirtuin 1) and RORα (retinoic acid receptor-related orphan receptor alpha) [114].

Emerging evidence indicates that IF modulates circadian and feeding rhythms [115]. An experimental study in mice has demonstrated that TRE attenuates body weight gain and enhances circadian rhythmicity in animals maintained on a high-fat diet [116]. Fasting regimens may also increase the amplitude of circadian oscillations. IF promotes hepatic glycogen depletion and subsequent lipolysis, leading to the production of the ketone body β-hydroxybutyrate (β-HB) [37]. Cignarella et al. demonstrated that IF can induce a two-fold increase in β-HB levels, under a 5:2 protocol [62]. β-HB functions as a signaling metabolite involved in the regulation of cellular functions and adaptive responses, including lipolysis, oxidative stress, and metabolic homeostasis [117]. Similarly, Zeb et al. found that TRE in a healthy male cohort enhanced circadian clock gene expression and improved metabolic function, with a significant increase in *BMAL1* and *CLOCK* gene expression levels compared with those in the control group [66]. Moreover, Xie et al. reported a significant increase in the mRNA expression of *BMAL1*, *PER2*, and *SIRT1* after TRE, specifically in eTRE, in a 5-week randomized controlled trial in healthy adults without obesity, supporting the ability of TRE to modulate circadian clock-related gene expression [69]. Furthermore, changes in gut microbiota diversity following TRE have been associated with *SIRT1* expression, particularly involving members of the *Prevotellaceae* and *Bacteroidetes*, further supporting interactions between the gut microbiota and circadian clock regulation [66,118]. Another mechanism by which TRE may also modulate circadian-driven processes is through downstream effects of mTOR pathway inhibition [119]. Kinases such as AMPK, CK1, and GSK3 regulate circadian clock components and play key roles in regulating the expression of *CRY1* and *CRY2* during fasting. In addition, the mTOR pathway contributes to the circadian phosphorylation of CREB, which can activate PER (circadian period protein) transcription.

The relationship between circadian rhythms, feeding behavior, and the gut microbiota is increasingly recognized as bidirectional. Feeding-fasting cycles constitute one of the strongest environmental signals synchronizing both peripheral circadian clocks and daily microbial oscillations. Conversely, the gut microbiota exhibits pronounced diurnal fluctuations in taxonomic composition, gene expression, and metabolic activity, which are disrupted by high-fat diets, irregular feeding schedules, or circadian misalignment [10,52]. Microbiota-derived metabolites, including SCFAs and bile acids, also display circadian oscillations and feedback to host tissues by modulating nutrient-sensing pathways, intestinal epithelial function, and peripheral clock gene expression, thereby reinforcing host circadian homeostasis [120,121]. These reciprocal interactions suggest that the metabolic benefits of IF may arise not only from fasting itself but also from the temporal alignment between nutrient intake, microbial rhythmicity, and host circadian physiology.

Collectively, these findings suggest that IF influences circadian regulation through coordinated effects on nutrient-sensing pathways, clock gene expression, and gut microbiota dynamics, although the precise molecular mechanisms remain to be fully elucidated. Because IF is tightly coupled to circadian regulation, the timing of microbiome sampling also emerges as a critical experimental variable. Feeding-fasting cycles drive rapid fluctuations in microbial abundance and function, indicating that microbial profiles obtained during fasting, feeding, or post-refeeding phases reflect distinct physiological states rather than directly comparable biological conditions.

## 4. Microbiota–Immune System Crosstalk: Effects of Intermittent Fasting

Bidirectional communication between the gut microbiota and host immune system is increasingly recognized as an important determinant of mucosal homeostasis and systemic inflammatory tone. IF imposes recurring cycles of nutrient deprivation and refeeding that reshape the microbial community structure and metabolic activity, thereby altering the repertoire of microbiota-derived molecules at the intestinal interface. These signals are integrated by epithelial and immune networks, positioning the gut as a central immunometabolic hub through which IF influences inflammatory tone and chronic low-grade inflammation.

A key mechanistic axis linking IF to immune regulation involves the modulation of constitutive innate immune activation driven by microbial products. As discussed earlier, chronic low-grade inflammation in cardiometabolic diseases has been linked to metabolic endotoxemia, whereby modest but sustained elevations in circulating lipopolysaccharide (LPS) engage pattern-recognition pathways and reinforcing a proinflammatory state [122]. In this context, IF protocols that attenuate the endotoxin burden provide a biologically plausible route for dampening innate immune activation. In adults with metabolic syndrome, an 8-week modified IF intervention was associated with gut microbiota shifts, accompanied by reductions in circulating LPS and inflammatory mediators, and improvements in cardiometabolic risk parameters [12]. These observations suggest that fasting-induced microbial alterations can align with immune-relevant systemic signatures rather than representing isolated compositional fluctuations.

Human studies integrating gut microbiome sequencing with immune phenotyping further indicate that fasting-based interventions are associated with changes in the gut microbiota and systemic immune profiles [62]. In patients with hypertension and metabolic syndrome, a short fasting intervention followed by controlled refeeding altered gut microbial taxa and metabolic pathways associated with SCFA production, while clinical responsiveness was associated with changes in circulating immune cell populations, including mucosa-associated invariant T (MAIT) cells, non-classical monocytes, and regulatory T cells (Tregs) [68]. Although these immune phenotypes are functionally linked to mucosal immune surveillance, they were measured in peripheral blood and therefore provide indirect evidence of immune responses that may be associated with microbiota remodeling rather than direct evidence of intestinal mucosal immune remodeling. These findings support the existence of coordinated microbiota–immune interactions during fasting but warrant confirmation through studies directly assessing intestinal immune compartments.

### 4.1. Innate and Adaptive Immune Mechanisms Mediated by Intermittent Fasting

At the intestinal barrier, innate immune circuits constitute the first layer of microbial surveillance. Mononuclear phagocytes integrate translocated microbial signals and shape the local cytokine profile, whereas innate lymphoid cells respond to microbial and dietary signals by promoting epithelial defense responses [123]. Type 3 innate lymphoid cells (ILC3) play a central role in barrier maintenance through the production of interleukin (IL)-22. In a recent preclinical mouse study, IF enhanced ILC3-derived IL-22 and was associated with downstream metabolic remodeling, suggesting that the ILC3-IL-22 axis may represent a fasting-responsive mucosal pathway linking intestinal immunity to systemic metabolism. However, this mechanism has thus far been demonstrated in a single animal study and requires validation in independent experimental models and human studies before its physiological relevance can be established [81]. More broadly, IL-22 is well recognized as a barrier-reinforcing cytokine that promotes epithelial antimicrobial defense and tissue repair [124], providing mechanistic insights into fasting-linked IL-22 induction in relation to intestinal containment.

Importantly, fasting-induced cytokine responses are unlikely to arise solely from intrinsic ILC3 activity; instead, they reflect a broader reorganization of the intestinal immune environment. In the same preclinical study previously described, IF restored communication among intestinal ILC subsets, dendritic cells, and macrophages in mice with obesity, which was accompanied by increased IL-22 production [81]. Although these findings provide mechanistic insight, their translational relevance remains uncertain because comparable evidence in humans is currently lacking. Transcriptomic analyses in the same study further suggested that IF may attenuate inflammatory signaling in dendritic cells by modulating pattern recognition pathways. Together, these findings highlight myeloid–ILC3 crosstalk as an additional layer through which fasting reshapes mucosal immune function at the epithelial interface. Moreover, macrophage polarization appears to contribute to the immunometabolic effects of IF. Preclinical studies suggest that fasting may promote a shift from inflammatory M1-like macrophages toward an anti-inflammatory M2-like phenotype, thereby reducing the production of TNF-α, IL-1β, and IL-6 while supporting tissue repair and metabolic homeostasis [125]. However, this mechanism has been demonstrated primarily in animal models and requires confirmation in human intervention studies.

In addition to the innate effector circuits, IF also modulates adaptive immune regulation. Among the T cell populations, the T helper (Th)17–Treg axis provides one of the clearest experimental links between fasting, microbiota remodeling, and immune regulation. In a mouse model of experimental autoimmune encephalomyelitis, IF increased microbial richness and altered microbial metabolic pathways, reduced IL-17-producing T cells and increased Treg cells in the gut. Notably, fecal microbiota transplantation from IF-treated donors conferred protection to recipient mice, supporting a causal contribution of the gut microbiota in mediating the adaptive immune effects of IF [62].

Another adaptive immune mechanism linking IF to microbiota–immune interactions is the regulation of secretory immunoglobulin A (sIgA), the predominant antibody isotype on mucosal surfaces. Lara-Padilla et al. demonstrated that IF altered sIgA levels and the number of IgA-producing plasma cells in the duodenum and ileum of stressed mice [75]. Moreover, IF has been reported to enhance IgA-associated bacterial clearance in enteric infection models [74]. Although human data remain limited, these findings suggest that IgA-mediated microbial containment is a potentially relevant pathway through which fasting may modulate microbiota–immune homeostasis.

### 4.2. Immunometabolic and Barrier-Related Outcomes of Intermittent Fasting

Beyond its effects on mucosal immunity, IF-driven microbial restructuring has also been linked to metabolic tissue adaptation, providing an additional dimension of microbiota-host crosstalk. In an ADF mice model with 24 h of fasting for 30 days, IF reshaped gut microbiota composition and increased fermentation products, such as acetate and lactate, which was coupled with robust beiging of white adipose tissue, and improvements in obesity, insulin resistance, and hepatic steatosis [11]. More recently, IF-induced increases in intestinal ILC3-derived IL-22 have been shown to directly signal to adipocytes and promote thermogenic programming, functionally linking fasting-responsive mucosal immunity to systemic metabolic remodeling [81].

These immune and metabolic adaptations ultimately converge on epithelial barrier integrity, which is a key determinant in the regulation of chronic low-grade inflammation. Barrier disruption increases systemic exposure to microbial products such as LPS, sustaining tonic innate activation and amplifying metabolic inflammation. Accordingly, IF interventions reporting reductions in circulating LPS or related markers support microbial translocation as a plausible mechanism contributing to the improved systemic inflammation in humans [12].

Nevertheless, barrier-related outcomes appear to be context-dependent, and temporal dynamics across the fasting and refeeding phases are likely to be critical. While several clinical and preclinical studies have associated IF with reduced circulating LPS and improvements in markers related to intestinal barrier function [12,42], these effects should not be generalized to all fasting regimens or physiological conditions. In a medically supervised 10-day Buchinger fasting study, the gut microbiota shifted toward a fasting-associated metabolic state, whereas systemic inflammatory mediators increased after food reintroduction, indicating that immune activation may transiently rebound during the refeeding phase rather than being uniformly suppressed throughout the intervention [63]. Moreover, recent preclinical evidence suggests that prolonged or inappropriate fasting regimens may adversely affect intestinal barrier homeostasis. In a long-term murine time-restricted feeding model (16:8 protocol), prolonged TRE impaired epithelial barrier integrity, reduced microbiota-derived indoleacrylic acid production, promoted Th17 cell differentiation, and exacerbated experimental colitis, whereas indoleacrylic acid supplementation partially reversed these effects [82]. Collectively, these findings indicate that the effects of IF on intestinal barrier function are highly dependent on the fasting protocol, host metabolic status, and disease context [30,42], emphasizing that IF should not be considered uniformly barrier-protective.

In summary, current evidence supports a coherent yet incomplete model in which IF reshapes microbial composition and metabolite activity and reduces endotoxin-linked innate stimulation in some clinical contexts. Moreover, IF is associated with immune adaptations that may contribute to epithelial barrier integrity and a shift toward regulatory immune states, although direct evidence of intestinal mucosal immune remodeling in humans remains limited. Current evidence shows that few human IF trials have simultaneously assessed functional intestinal permeability, high-resolution functional microbiome profiling, and mucosal immune cell populations. Addressing this gap will require regimen-specific studies that incorporate microbial translocation markers, metabolomics, IgA profiling, targeted immunophenotyping of lamina propria myeloid subsets, ILC3 cytokine responses, and Th17/Treg balance.

## 5. Effects of Intermittent Fasting on Gut Microbial Regulation in Cardiometabolic Disorders

The effects of IF on gut microbiota have important implications for cardiometabolic health. In this section, we highlight findings that specifically extend the discussion beyond the general microbiota shifts described above, focusing on obesity-related lipid metabolism and glycemic control.

### 5.1. Obesity and Lipid Metabolism

The pathophysiology of obesity has been associated with a dysbiotic state characterized by alterations in gut microbiota composition and function [126]. In preclinical models, ADF has been shown to influence energy metabolism through a gut microbiota-adipose tissue axis. Li et al. reported that a 24 h fasting ADF protocol for 30 days activates beige adipose tissue thermogenesis, along with marked changes in the relative abundance of major bacterial phyla and an increased Firmicutes/Bacteroidetes ratio [11]. Metabolomic and metagenomic analyses further indicated a shift toward lactate and acetate-producing taxa, with higher circulating levels of these metabolites, suggesting a link between microbial remodeling and white adipose tissue browning [11]. Likewise, in mice fed a high-fat diet, ADF was associated with enrichment of *Allobaculum*, a genus linked to glucose metabolism and SCFA production [79]. By contrast, clinical studies of 12-week TRE (16:8) intervention in individuals with overweight or obesity have reported no significant changes in overall microbiota diversity or in the Firmicutes/Bacteroidetes ratio, suggesting that the metabolic impact of TRE may depend on intervention duration, study population and/or baseline metabolic status [59,60].

Beyond compositional changes, IF may also influence lipid metabolism through circadian regulation in the intestinal epithelium. NFIL3 (nuclear factor interleukin-3 regulated), a clock-controlled transcription factor, regulates rhythmic expression of genes involved in lipid uptake, processing, and storage, and its oscillations are shaped by microbiota-dependent signaling pathways [121]. Similarly, HDAC3 (histone deacetylase 3), coordinates circadian histone acetylation in intestinal epithelial cells and contributes to synchronized nutrient handling and lipid absorption [127]. Collectively, these findings suggest that IF may modulate lipid metabolism not only through microbial remodeling, but also through circadian-dependent host pathways, although direct evidence in fasting interventions remains limited.

### 5.2. Diabetes and Glycemic Control

Evidence indicates that gut microbiota composition is altered in patients with impaired glucose tolerance and T2DM [128]. In animal models, long-term ADF has been associated with microbial changes that may contribute to improved gut barrier function and metabolic outcomes [76]. These findings are consistent with clinical trials showing that IF can lower blood glucose levels and improve glycemic control in patients with T2DM [42,76,129,130].

The gut microbiome also dynamically responds to fasting and refeeding cycles, suggesting a role in the regulation of glucose homeostasis [131,132]. In mice, *Lactobacillus* has been reported to predominate during fasting, whereas *A. muciniphila* is markedly enriched during the feeding period [77]. Importantly, *A. muciniphila* has been inversely associated with blood glucose levels in both rodents and humans [133]. Although nutrient ingestion remains the primary physiological stimulus for GLP-1 secretion from enteroendocrine L cells, increasing evidence suggests that *A. muciniphila* and its metabolites may modulate this response [133,134]. Mechanistic studies indicate that microbial-derived metabolites, including propionate, together with *A. muciniphila*-derived proteins P9 and Amuc-1100, may enhance nutrient-induced GLP-1 secretion or increase L-cell responsiveness through indirect signaling pathways rather than acting as the primary trigger of hormone release [135]. Together, these observations support the concept that nutritional timing may influence glycemic control through coordinated effects on the gut microbiota, enteroendocrine signaling, and host metabolic responses, although further mechanistic studies in humans are needed.

## 6. Alternative Dietary Strategies During Intermittent Fasting to Enhance Gut Microbiota

It remains unclear whether improving dietary quality during the feeding window is sufficient to modulate or enhance the metabolic effects of IF. However, diet is one of the main factors shaping the composition and function of the gut microbiota. Therefore, the foods consumed during the eating window may influence gut microbiota and, consequently, the metabolic response to IF. In this section, we discuss the current evidence on dietary fiber, prebiotics, and probiotics as nutritional strategies that may complement IF by modulating gut microbiota composition and function.

*Prebiotics and high-fiber diets.* Dietary fiber has been proposed as a nutritional strategy that may facilitate the metabolic switch associated with fasting by improving glycemic control and insulin sensitivity, predominantly through modulation of gut microbiota [136]. Fermentable fibers promote the production of SCFAs, which play an important role in glucose regulation, lipid metabolism, and inflammatory signaling pathways [137]. Therefore, the combination of IF with dietary fiber or prebiotics may exert complementary effects on microbial ecology and host metabolic outcomes. Although clinical evidence remains limited, available studies support these interactions. In a human intervention study, inulin supplementation during fasting increased the abundance of *Bifidobacterium* spp. and produced a gut microbial profile distinct from that observed with fasting alone [138]. Preclinical studies have further reinforced this concept. In mice fed a high-fat diet, the combination of IF with insoluble dietary fiber derived from okara resulted in greater improvements in dyslipidemia and glucose homeostasis than IF alone [139]. These metabolic benefits were accompanied by restoration of gut microbial composition and normalization of SCFA production. Similarly, research conducted in diabetic mouse models has shown that the combined administration of IF with prebiotic dietary fibers restored microbial diversity and corrected alterations in specific bacterial taxa [140]. Overall, these findings indicate that incorporating fiber or fiber-like prebiotics into fasting regimens may enhance microbiota resilience and optimize metabolic regulatory pathways.

*Probiotics.* Probiotics are defined as live microorganisms that, when administered in adequate amounts, confer measurable health benefits to the host [141]. Owing to their ability to modulate gut microbiota composition and function, probiotics have been proposed as a complementary nutritional strategy to IF. In addition to promoting gastrointestinal homeostasis, probiotics may influence host metabolism and immune responses, suggesting that their combination with IF could enhance some of the metabolic and immunological adaptations associated with fasting [142,143,144]. Moreover, some evidence suggests that probiotics may influence hormones related to appetite and satiety, which may help individuals adhere to their fasting protocols more easily and manage their hunger [145]. A recent study assessed the potential synergy between IF and supplementation with *Lacticaseibacillus rhamnosus* probiotic in individuals with prediabetes, aiming to reduce glycated hemoglobin (HbA1c) levels. These results suggest that the combination of IF and probiotic supplementation not only aids in weight loss and glycemic improvement but also offers additional physiological benefits compared with IF alone [146].

Current evidence, primarily derived from preclinical models, suggests that IF may increase the abundance of *A. muciniphila* and *F. prausnitzii*, restore circadian rhythmicity, and enhance the production of SCFAs and secondary bile acid metabolites [147]. In parallel, multi-strain probiotic supplementation has been shown to independently reduce fasting glucose and HbA1c levels. Both interventions appear to converge mechanistically by attenuating metabolic endotoxemia and improving intestinal barrier integrity [147]. Animal studies further suggest that combining IF with SCD (symbiotic culture of bacteria and yeast) probiotic supplementation enhances these effects, improving intestinal integrity while reducing intestinal inflammation [148]. Specifically, this combined intervention reduced mast cell density and the expression of pro-inflammatory markers, including TNF-α and NF-κB, in both ileal and colonic tissues [148]. At the hepatic level, the combination of IF and SCD-based probiotic supplementation significantly reduced cholesterol ester accumulation, improved protein phosphorylation patterns, and attenuated histopathological features of liver injury such as hepatocyte degeneration, lymphocytic infiltration, steatosis and fibrosis [149]. These findings suggest that probiotic supplementation may enhance the hepatoprotective effects of IF [149]. Similarly, a recent study investigated the effects of combining probiotic-enriched camel milk supplementation with IF over a six-week period in an obese rat model. The findings indicated that this combined intervention was associated with greater weight loss, significant improvements in metabolic parameters, and partial reversal of obesity-related histopathological changes [150]. Based on these outcomes, the authors suggested that probiotic camel milk administered alongside IF may represent a promising adjunctive strategy for facilitating weight reduction, enhancing antioxidant capacity, and reducing the risk of metabolic complications associated with obesity [150]. In addition, this intervention increased gut microbial diversity and promoted favorable shifts in microbial community composition, changes that may contribute to the observed metabolic improvements [151].

Although combining IF with probiotics or prebiotics represents a promising strategy to enhance gut microbiota modulation, the current clinical evidence is limited. Most available human studies include relatively small sample sizes, short intervention periods, and considerable heterogeneity in fasting regimens, probiotics, strains, dosages, and participant characteristics, making direct comparisons challenging and limiting the generalizability of the findings [152,153]. Importantly, the biological effects of probiotics are strain-specific and should not be extrapolated across different bacterial species or formulations [154]. Furthermore, interindividual variability in baseline gut microbiota composition, dietary habits, and metabolic status may substantially influence the response to probiotic supplementation, contributing to the inconsistent outcomes reported across clinical studies [25,152]. Therefore, although preclinical evidence supports potential synergistic interactions between IF and microbiota-targeted interventions, robust clinical evidence remains insufficient. Future randomized controlled trials using standardized fasting protocols, well-characterized probiotics strains, and comprehensive functional microbiome analyses are needed before definitive clinical recommendations can be established [152,154].

## 7. Discussion

A growing body of evidence supports IF as a promising dietary strategy for weight management and the improvement of metabolic health [38]. Beyond its potential role in obesity management, IF has been associated with multiple physiological benefits in the host, including improvements in cardiometabolic health, anti-aging processes, antioxidant activity, and neuroprotective effects [3].

Current findings indicate that IF can influence gut microbiota diversity and composition; however, considerable heterogeneity exists in the reported taxonomic changes across studies [6,8,11,58,64,65,66]. The specific microbial taxa affected by IF often differ between studies, probably owing to differences in fasting protocols, study populations, intervention duration, dietary background, and microbiome assessment methods. Therefore, greater standardization of study design and microbiome analysis protocols in IF research will be essential to facilitate comparisons across studies and identify reproducible microbial signatures.

Beyond compositional changes, IF has also been associated with alterations in gut microbiota-derived metabolites, inflammatory markers, intestinal barrier function, and circadian regulation, suggesting that the gut microbiota may contribute to some of the metabolic and immunological effects of fasting [12,20,122,155,156,157,158,159]. However, despite these promising findings, the mechanistic links between IF-induced microbial changes and host metabolic outcomes remain incompletely understood.

In spite of increasing research in this area, several limitations remain. IF protocols are highly heterogeneous, and their long-term effects remain debated despite promising findings [8]. From a microbiome perspective, some studies are limited by the availability of microbial datasets and analytical approaches [6]. Furthermore, most studies rely primarily on fecal samples, often overlooking regional differences in the composition and function of the gut microbiota [8]. In addition, methodological heterogeneity in microbiome profiling further limits direct comparisons across studies. Most available studies rely on 16S rRNA gene sequencing; however, important differences exist in DNA extraction protocols, targeted hypervariable regions (e.g., V3–V4 vs. V4), sequencing platforms, sequencing depth, bioinformatic pipelines, taxonomic reference databases, and statistical normalization methods. These methodological differences can substantially influence taxonomic assignments, diversity estimates, and the detection of differentially abundant taxa, thereby contributing to variability in reported microbiota signatures independent of the fasting intervention itself [87,160,161].

Technical variability between studies, together with differences in dietary control and participant characteristics, may contribute to inconsistent findings and hinder cross-study comparisons [8]. An additional methodological factor contributing to the heterogeneity of current evidence is the timing of fecal sample collection relative to fasting-refeeding cycles. Gut microbial communities exhibit pronounced temporal oscillations driven by feeding behavior and circadian rhythms [10,162]. Consequently, microbial abundance measured during fasting, active feeding, or post-refeeding phases may differ substantially, even within the same intervention. Therefore, taxonomic changes reported across studies may not be directly compared when sampling timepoints are not standardized [52,163]. Future studies should carefully harmonize sample collection relative to fasting duration, refeeding, circadian timing, and the exact time point of sample collection. Standardizing these methodological aspects would improve reproducibility and facilitate the identification of consistent microbial signatures associated with IF across different studies.

Another important limitation that should be considered is the potential for publication bias within the current IF literature. Studies reporting significant improvements in gut microbiota composition, microbial metabolites, or metabolic outcomes are more likely to be published than those reporting neutral or negative findings, potentially overestimating the consistency and magnitude of the beneficial effects attributed to IF [164,165]. In addition, substantial heterogeneity in fasting protocols, microbiome sequencing methodologies, bioinformatic pipelines, and outcome reporting further complicates the interpretation and reproducibility of the available evidence [87,166]. Future systematic reviews should formally assess publication bias whenever feasible, whereas adequately powered randomized controlled trials with standardized microbiome methodologies and transparent reporting of both positive and negative findings will be essential to provide a more balanced understanding of the effects of IF on the gut microbiota.

Moreover, relatively few studies have integrated microbiome, metabolomic, and host phenotypic data, limiting the ability to determine whether microbial alterations are causally involved in the physiological effects of IF or simply occur in parallel with other fasting-induced adaptations. In addition, human variability, such as ethnic, geographic, and demographic factors, as well as individual characteristics including body mass index, sex, and age, may limit the generalizability of conclusions regarding microbiome alterations [167]. Nevertheless, inter-individual microbiome heterogeneity may also represent an opportunity to identify personalized responses to different IF interventions, highlighting its potential as a key strategy for precision nutrition [25].

Mechanistic insights into IF-induced gut microbiota modulation may help explain both the observed phenotypic effects and interindividual variability in responses to IF protocols under similar dietary conditions [168]. However, important differences in gut microbiota composition and microbial responses to IF exist between humans and animal models, highlighting the need for caution when extrapolating mechanistic findings across species [34]. IF promotes the production of microbial metabolites that may exert downstream metabolic effects on host metabolic physiology and disease processes [11]. Although these findings are not specific to IF, they support the concept that modulation of microbial metabolic activity, particularly SCFAs production and signaling, constitutes a common mechanism linking gut microbiota remodeling with host metabolic regulation across different intervention modalities. Within this framework, IF may represent one of several interventions capable of engaging this microbiota-dependent metabolic axis. For instance, SCFA production has been associated with beneficial effects on adipose tissue browning, energy expenditure, GLP-1 secretion by intestinal L cells, and improved glycemic control [100]. Secondary bile acids have also been shown to exert protective effects against hypertension, diabetic retinopathy, and cognitive impairment [169]. Nevertheless, changes in the abundance of specific microbial taxa should not be interpreted as direct evidence of altered metabolic function, underscoring the importance of integrating taxonomic, functional, and metabolomic analyses to better understand the mechanisms underlying the physiological effects of IF.

As previously discussed, bidirectional crosstalk between gut microbiota and the host immune system plays a central role in maintaining mucosal homeostasis and regulating systemic inflammation [25]. Evidence suggests that IF may attenuate inflammation by reducing metabolic endotoxemia and the levels of circulating inflammatory mediators [122]. These microbial alterations may be associated with the modulation of both innate and adaptive immune pathways, promoting a shift toward regulatory immune states with downstream consequences for metabolic tissues, such as adipose tissue and the liver [12]. Nevertheless, the causal contribution of these microbial changes to the immunological effects of IF remains to be established and further mechanistic studies are needed to elucidate the underlying molecular and biochemical pathways.

Collectively, current evidence suggests that the metabolic and microbiota-related benefits of IF may be further enhanced by targeted dietary strategies implemented during the feeding window [1]. In particular, diets enriched with fermentable fibers and prebiotics appear to enhance microbial diversity and promote the production of SCFAs [170,171]. Experimental and early clinical studies have indicated that combining IF with fiber supplementation can restore microbial balance and improve metabolic parameters, such as insulin sensitivity and lipid profiles [140]. Likewise, probiotic supplementation has been proposed as a complementary strategy for reinforcing gut microbiota stability, improving intestinal barrier integrity, and attenuating inflammatory signaling pathways. Preclinical and limited human evidence suggest that the combined implementation of IF and probiotics may exert synergistic effects on metabolic regulation, immune homeostasis, and tissue integrity, including improvements in glycemic control, reductions in metabolic endotoxemia, and hepatoprotective effects [146]. However, evidence from human studies remains limited. Therefore, future studies should investigate whether combining IF with probiotics or prebiotics provides additional benefits over either intervention alone in adequately powered clinical trials.

Although IF has shown promising metabolic and microbiota-related benefits, its implementation should be individualized and interpreted with caution. Current evidence indicates that IF may not be appropriate for all populations, particularly pregnant or lactating women, children and adolescents, frail older adults at risk of malnutrition or sarcopenia, individuals with current or previous eating disorders, and patients receiving insulin or sulfonylureas, in whom fasting may increase the risk of hypoglycemia or nutritional deficiencies [38,42,172]. Moreover, the long-term safety and efficacy of IF remain insufficiently established in several clinical populations, as most interventions studies have been relatively short and have enrolled carefully selected participants [44,173]. In addition, considerable interindividual variability exists in the physiological response to IF. Factors including age, sex, ethnicity, baseline microbiota composition, habitual dietary patterns, metabolic status, chronotype, and genetic background may all influence microbial remodeling and cardiometabolic outcomes following fasting interventions [25,84,86]. From a clinical perspective, IF should be considered a complementary nutritional strategy rather than a universal therapeutic approach. Its implementation should be individualized according to the patient’s metabolic status, comorbidities, lifestyle, and treatment goals. At present, available evidence does not support recommending one specific IF protocol for microbiota modulation, highlighting the need for personalized approaches and adequately powered clinical trials [30,42].

A major challenge in understanding the interaction between IF and the gut microbiota is that most current evidence is derived from taxonomic profiling, which provides limited insight into microbial function. Although high-throughput sequencing has advanced our knowledge of IF-associated changes in microbial composition, alterations in taxonomic abundance cannot be directly translated into functional or metabolic consequences. This limitation hinders the identification of the microbial pathways and metabolites that may mediate host responses to fasting. Future studies should therefore move beyond descriptive taxonomic analyses by integrating strain-level characterization with metagenomics, transcriptomics, metabolomics, and other functional approaches. Such integrative strategies will be essential to determine whether specific microbial taxa, their metabolic activities, or both are responsible for the physiological effects attributed to IF. In this context, microorganisms such as *A. muciniphila*, which have repeatedly been associated with metabolic health, may represent promising candidates for mechanistic investigation.

Finally, establishing causal links between microbial function and host metabolism will be critical for translating microbiome research into precision nutrition strategies and personalized dietary interventions.

## 8. Conclusions

**Figure 2:**
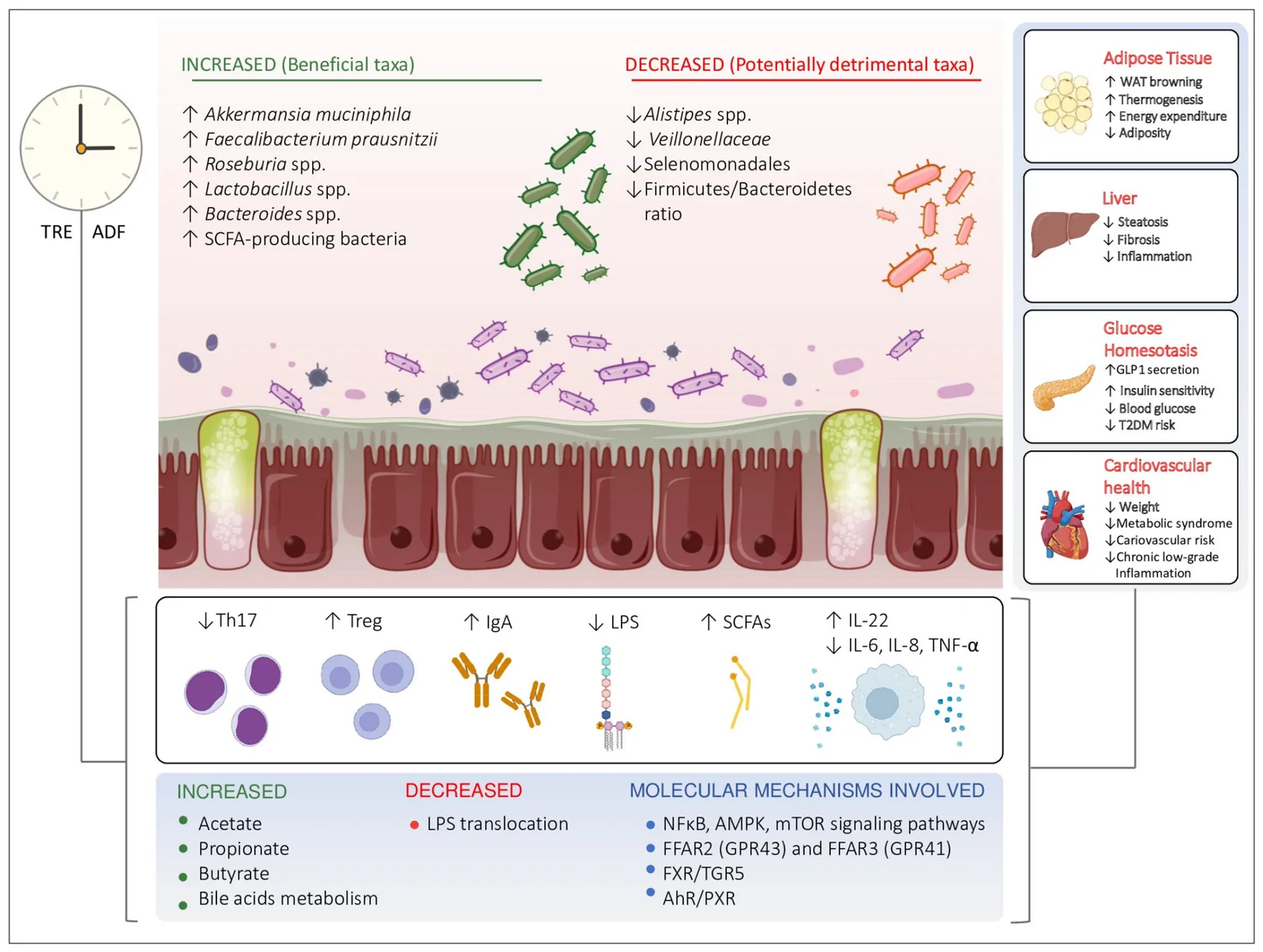
Proposed mechanisms linking intermittent fasting (IF), gut microbiota remodeling, and metabolic health. Time-restricted eating (TRE) and alternate-day fasting (ADF) reshape the composition and metabolic activity of the gut microbiota, promoting the production of beneficial microbial metabolites, including short-chain fatty acids (SCFAs). These microbial adaptations contribute to the preservation of intestinal barrier integrity, reduced lipopolysaccharide (LPS) translocation and systemic inflammation, and improved host metabolic homeostasis. Through these mechanisms, IF may enhance adipose tissue function, hepatic metabolism, glucose regulation, and cardiovascular health. GLP-1, glucagon-like peptide-1; IL, interleukin; T2DM, type 2 diabetes mellitus; WAT, white adipose tissue.

IF has emerged as a promising nutritional strategy capable of improving metabolic health through mechanisms that extend beyond simple caloric restriction. Growing evidence indicates that modulation of the gut microbiota represents one of several mechanisms through which IF may exert its beneficial metabolic effects. Fasting-induced changes in feeding patterns and nutrient availability can reshape microbial diversity, composition, and metabolic activity, favoring the enrichment of taxa such as *A. muciniphila*, *F. prausnitzii*, and other SCFA-producing bacteria. These microbial shifts may contribute to the production of bioactive metabolites that regulate energy homeostasis, glucose metabolism, intestinal barrier integrity, and inflammatory signaling (Figure 2).

In addition, IF interacts with circadian regulation and immune pathways, further supporting the concept of a dynamic microbiota–immune–metabolic axis involved in host adaptation to fasting. Through these interconnected mechanisms, IF may contribute to improving cardiometabolic health, including weight loss, insulin sensitivity, and glycemic control, while also promoting metabolic resilience and reducing chronic low-grade inflammation (Figure 2).

Despite these promising findings, important challenges remain. The heterogeneity of fasting protocols, interindividual variability in gut microbiome profiles, and the predominance of preclinical models limit the interpretation and generalizability of current findings. Future research should prioritize well-designed longitudinal clinical studies integrating microbiome profiling, metabolomics, and immunological assessments to elucidate underlying causal mechanisms. Such integrative approaches will be critical for advancing precision nutrition strategies and optimizing microbiota-targeted interventions based on IF interventions.

## Figures and Tables

**Table 1:** Approaches and characteristics of intermittent fasting.

Protocol	Frequency	Duration (Fasting/Restriction Period)	Eating Window	Energy Intake (Fasting/Restriction Period)
ADF (Alternate Day Fasting)	Every other day	24 h	24 h (ad libitum on feeding days)	Strictly controlled energy intake (≈25% of usual intake, ≈500 kcal)
PF (Periodic Fasting; 5:2)	Two days weekly	24 h each fasting day	24 h (ad libitum on feeding days)	Strictly controlled energy intake (500–800 kcal)
TRE (Time-Restricted Eating)	Every day	14–18 h	6–10 h (ad libitum)	No energy intake

## Data Availability

No new data were created or analyzed in this study. Data sharing is not applicable to this article.

## Abbreviations

The following abbreviations are used in this manuscript:

ADF	Alternate-day fasting
AhR	Aryl hydrocarbon receptor
BCAA	Branched-chain amino acids
BMAL1	Brain and muscle ARNT-like protein 1
CLOCK	Circadian locomotor output cycles kaput
CR	Caloric restriction
GLP-1	Glucagon-like peptide-1
IAA	Indole-3-acetic acid
IAId	Indole-3-aldehyde
IF	Intermittent fasting
ILC3	Type 3 innate lymphoid cells
IPA	Indole-3-propionic acid
NFIL3	Nuclear factor interleukin-3 regulated
PF	Periodic fasting
PXR	Pregnane X receptor
PYY	Peptide YY
SCD	Symbiotic culture of bacteria and yeast
SCFAs	Short-chain fatty acids
T2DM	Type 2 diabetes mellitus
TMAO	Trimethylamine *N*-oxide
TRE	Time-restricted eating
